# An updated analysis of variations in SARS-CoV-2 genome

**DOI:** 10.3906/biy-2005-111

**Published:** 2020-06-21

**Authors:** Osman Mutluhan UGUREL, Oguz ATA, Dilek TURGUT-BALIK

**Affiliations:** 1 Department of Bioengineering, Faculty of Chemical and Metallurgical Engineering, Yıldız Technical University, İstanbul Turkey; 2 Department of Basic Sciences, School of Engineering and Natural Sciences, Altınbaş University, İstanbul Turkey; 3 Department of Software Engineering, School of Engineering and Natural Sciences, Altınbaş University, İstanbul Turkey

**Keywords:** SARS-CoV-2, COVID-19, genomic diversity, codon bias, multiple sequence alignment

## Abstract

A novel pathogen, named SARS-CoV-2, has caused an unprecedented worldwide pandemic in the first half of 2020. As the SARS-CoV-2 genome sequences have become available, one of the important focus of scientists has become tracking variations in the viral genome. In this study, 30366 SARS-CoV-2 isolate genomes were aligned using the software developed by our group (ODOTool) and 11 variations in SARS-CoV-2 genome over 10% of whole isolates were discussed. Results indicated that, frequency rates of these 11 variations change between 3.56%–88.44 % and these rates differ greatly depending on the continents they have been reported. Despite some variations being in low frequency rate in some continents, C14408T and A23403G variations on Nsp12 and S protein, respectively, observed to be the most prominent variations all over the world, in general, and both cause missense mutations. It is also notable that most of isolates carry C14408T and A23403 variations simultaneously and also nearly all isolates carrying the G25563T variation on ORF3a, also carry C14408T and A23403 variations, although their location distributions are not similar. All these data should be considered towards development of vaccine and antiviral treatment strategies as well as tracing diversity of virus in all over the world.

## 1. Introduction

COVID-19 (novel COronaVIrus Disease 2019) is caused by a novel pathogen that is pursued closely by all over the world after the worldwide pandemic was declared by WHO on March 11th. It has become one of the most important health problems by causing nearly 5M confirmed cases and over 300K deaths from 213 countries/regions in a couple of months (WHO, 2020a)^1^1WHO (2020a). Coronavirus disease (COVID-2019) situation reports. [online]. Website: https://www.who.int/emergencies/diseases/novel-coronavirus-2019/situation-reports accessed 17 May 2020].. More than 17,000 papers by the query “COVID-19” indexed in PubMed/NCBI and nearly 200 of them, as of 29th May 2020, about genome of severe acute respiratory syndrome-COronaVirus-2 (SARS-CoV-2) which is the agent responsible for the disease. 

### 1.1. Taxonomy of SARS-CoV-2

SARS-CoV-2, is an enveloped, +ssRNA virus belonging to the Coronaviridae family that are classified into 4 major genera: Alphacoronavirus, Betacoronavirus, Gammacoronavirus, and Deltacoronavirus by phylogenetic studies and classified in the Betacoronavirus genus, Sarbecovirus and grouped as SARS-Like CoVs (Gorblenya et al., 2020). CoVs can infect many of animal species from different genus including mammals, avians and reptiles (Gorblenya et al., 2006; Wu et al., 2020; Gorblenya et al., 2020). Until December 2019, there were 6 coronavirus species known as human pathogen. Four of them (229E, OC43, NL63, and HKU1) have caused common cold and others 2 worldwide outbreaks (SARS 2003; MERS 2012) over the last 20 years (Su et al., 2016; Zhu et al., 2020). Recently, a 7th coronavirus species has been discovered in December 2019 and first named as 2019 novel Coronavirus (2019 nCoV) and then as SARS-CoV-2 (Gorblenya et al., 2020).

### 1.2. The outbreaks of CoVs

An outbreak of severe acute respiratory syndrome (SARS) was reported in November 2002. Despite patients carried symptoms of a viral infection, no pathogen causing pneumonia was identified, and in a few months, it was revealed that a novel CoV had caused this syndrome (Peiris et al., 2003). The filiation studies about SARS showed that the early cases were mostly seen among restaurant workers in Guandong Province, China and this information led researchers to suspect that transmission source of the virus might be an animal like bat or civet, that frequently consumed in that province (Zhong et al., 2003; Guan et al., 2003; Su et al., 2016). This was the first-known CoV outbreak that caused 8096 cases and 774 deaths in 37 countries or areas until July 2003 (WHO, 2004)^2^2WHO (2004). Summary of probable SARS cases with onset of illness from 1 November 2002 to 31 July 2003. [online]. Website: https://www.who.int/csr/sars/country/table2004_04_21/en/ [accessed 17 May 2020]..

In the summer of 2012, 9 years after SARS outbreak was controlled, a novel coronavirus disease called Middle East Respiratory Syndrome (MERS) was reported in Saudi Arabia (WHO, 2019)^3^3WHO (2019). Middle East respiratory syndrome coronavirus (MERS-CoV). [online]. Website: https://www.who.int/en/news-room/fact-sheets/detail/middle-east-respiratory-syndrome-coronavirus-(mers-cov) [accessed 17 May 2020].. A 60-year-old man with severe fever and cough, was diagnosed as pneumoniae and, as in SARS, no pathogen causing pneumonia was detected, however, fragments that amplified from some of PCR assays for the detection of coronaviruses were sequenced and analysis of the results indicated a novel coronavirus named MERS-CoV relative with HKU4 and HKU5 (Zaki et al., 2012; de Groott al., 2013; Yin and Wunderink, 2013). Despite the transmission from animal to human has not been clearly verified, the dromedary camels have been proposed as the main reservoir host for the virus (Azhar et al., 2014; WHO, 2019). This epidemic was mostly effective in Saudi Arabia and in the gulf countries, caused nearly 2500 confirmed cases with 35% death rate (WHO, 2019). 

Transmission mechanism of both SARS-CoV and MERS-CoV from animal to human was reported to be the direct contact with host animals or consumption of raw milk, meat or urine (Yin and Wunderink, 2013; WHO, 2019). The studies listed the 3 major causes of SARS epidemic termination: the public health strategies, scientific development in biology and medicine, and hygiene practices (Chew, 2007). 

### 1.3. Evolution of SARS-CoV-2

It was already known that the Spike (S) glycoprotein plays a determining role in CoV infections (Sanchez et al., 1999) and is effective in viral entry and pathogenesis (Gallagher and Buchmeiert, 2001). During the ongoing SARS outbreak, it has been discovered that the novel virus has developed a new viral entry mechanism by binding S protein to angiotensin-converting enzyme 2 (ACE2) receptors (Li et al., 2003), as the identification mark of SARS-CoV adaptation which has been caused by the mutations on S protein residues between 318–510 that named as receptor binding protein (RBD) (Wong et al., 2004; Li et al., 2005). After the SARS outbreak, animals such as bats, civets, and pangolins, have been seen as the main reservoir hosts of the SARS-related CoVs that has similar entry mechanism and scientists have focused on studying viruses hosted by these animals (Ge et al., 2013; Li, 2016; Cui et al., 2019; Liu et al., 2019). Despite RBD of MERS-CoV-2 binds to dipeptidyl peptidase 4 (Raj et al.,2013), it has been revealed that RBD of SARS-CoV-2 binds ACE2, like in SARS-CoV (Tai et al., 2020). Although it has been lasted about 1 year to resolve this mechanism for the SARS-CoV (Li et al., 2003), this timing was much shorter, as nearly 3 months, with the research ability that have been gained with the developing DNA sequencing technologies and over years of experience in this area (Tai et al., 2020; Ou et al., 2020). 

### 1.4. Genomic studies on CoVs

There are more than 7,000 complete genome entry uploaded to Nucleotide/NCBI databases from Coronaviridae family between 2002–2020, more than half of them is being SARS-CoV-2 (www.ncbi.nlm.nih.gov/nuccore/, accessed 29th May 2020) and it is increasing in parallel to the development of sequencing technology. The coronavirus genome structure has been characterised by various studies (Kocherhans et al., 2001; Brian and Baric, 2005; Gorblenya et al., 2006; Yang and Leibowitz, 2015; Madhugiri et al., 2016) with the genome size around 30kb. CoVs are referred to have the largest known genome size among RNA viruses (Brian and Baric, 2005). All CoV genomes contain a large gene region (named ORF) encoding nonstructural proteins (Nsp) which are responsible for mostly replication and the genes encoding spike (S) glycoprotein, envelope (E) protein, membrane (M) glycoprotein and nucleocapsid (N) protein have also been found in common (Brian and Baric, 2005; Gorblenya et al., 2006). 

The genome sequence of SARS-CoV-2 was first characterised by Wu et al. in December 2019 (Wu et al, 2020) and appointed as reference genome of SARS-CoV-2 (NC_045512.2; 1-29870); consist of 11 gene regions; ORF1ab (266-21555), S (21563-25384), ORF3a (25393-26220), E (26245-26472), M (26523-27191), ORF6 (27202-27387), ORF7a (27394-27759), ORF7b (27756-27887), ORF8 (27894-28259), N (8274-29533), ORF10 (29558-29674) by NCBI (Genbank, 2020). 

Although this reference genome sequence is commonly used in most studies, it is extremely important to monitor the variations in the virus genome to understand the evolution and spread of the virus and also to use this information in the development possible treatments and vaccines accordingly. For this purpose, GISAID (GISAID, 2020; Elbe and Buckland-Merrett, 2017; Shu, and McCauley, 2017) which collects and shares genome sequences and related clinical/epidemiological data for monitoring annual influenza strains, established a new database called EpiCoV and the first SARS-CoV-2 genome was shared on 10th January 2020. More than 30,000 genome sequences of SARS-CoV-2 were uploaded to EpiCoV databases between 10th January and 20th May 2020 (EpiCoV, 2020)^4^4EpiCoV (2020). Pandemic coronavirus causing COVID-19 [online]. Website: https://www.epicov.org/ [accessed 17 May 2020].. 

In this study; we have used these data to analyse the mutations on SARS-CoV-2 genome using a software based on multiple sequence alignment (Strategy Based Local Alignment Tool: ODOTool) that have been originally developed for bacterial SNP determination in our studies. Now, we targeted to analyse the mutations that have emerged in at least 10% of SARS-CoV-2 genomes in all 30366 sequences submitted in GISAID by May 20th, 2020 using the ODOTool in terms of date and location they occurred, the relationship with each other and their effect on the primary protein structure.

## 2. Materials and methods 

### 2.1. Data optimization and development of strategy based local alignment tool: ODOTool

Despite the Strategy Based Local Alignment Tool (ODOTool) used in this study was originally developed by our group for bacterial single nucleotide polymorphism (SNP) determination, it was reasonable to test the abilities of the tool using a different dataset with the emergence of SARS-CoV-2 causing COVID-19 pandemic and this is applied in the present study to analyse variations in viral genome. 

The first genome sequence of SARS-CoV-2 has been identified and submitted with the accession number of MN908947.1 to Genbank by Wu et al. in December 2020 (Wu et al., 2020), then curated by NCBI staff, reviewed by RefSeq (O’Leary et al., 2015), and appointed as the reference genome of SARS-CoV-2 (NC_045512.2) (Genbank, 2020). Genomic sequences (30366) of SARS-CoV-2 isolates submitted on GISAID/ EpiCoV database by 21st May 2020, have been downloaded in FASTA file format, into our local database for alignment and analysis.

**Figure 1:**
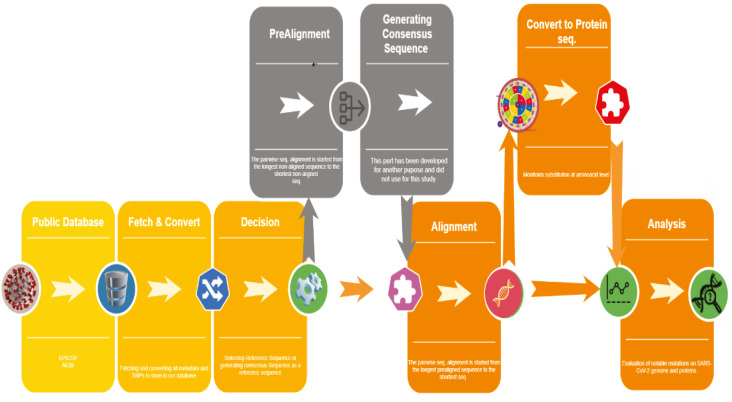
Workflow of ODOTool.

Workflow of ODOTool is given in Figure 1. The main purpose of developing the ODOTool was analysing bacterial gene sequences for single nucleotide polymorphism (SNP) determination. It was designed to download bulk data of gene sequences from open access databases such as Genbank/NCBI and EMBL-EBI, to prepare data first for prealignment and then for alignment and analysis of the aligned sequences. Prealignment and generating universal consensus sequences module (shown in grey in Figure 1) is an additional step for analysis of the sequences among different genus, hence it is not used in the present study.

The ODOTool has been coded with Python programming language by Biophyton library (Cock et al., 2009). After the genome sequence downloading process, all data were stored in our local database with annotations such as isolate name, location, collection, and submission dates etc. The data downloaded from different databases were not in a standard format. Therefore, an additional data conversion step was applied to remove any undesired features or information like FASTA comments, line feeds, blank spaces and all sequences set to upper cases etc. to standardise all data prior to further analyses. Modified Needleman-Wunsch algorithm, modified BLOSUM 62 scoring matrix and adjusted gap penalties (match = 4, mismatch = –1, reference genome open gap score = –1000, reference genome extend gap score = –1000, isolate genome open gap score = –20, isolate genome extend gap score = –4) were used for alignment step. Aligned nucleotide sequences were then converted to protein sequences to determine missense mutations using standard codon table (Peabody, 1989). Aligned nucleotide and protein sequences of all downloaded isolate genomes were stored in local database for computational, visual, and statistical analysis.

The alignments performed by ODOTool were validated with multiple alignment program MAFFT v7 (Kuraku et al., 2013; Katoh et al., 2017) to evaluate the accuracy and performance of ODOTool. Data size suggested for accuracy by MAFFT v7 is 200 genome sequences . Therefore, 200 SARS-CoV-2 genomes were randomly selected and the same sequences were analysed by using both MAFFT v7 and ODOTool. The genomic positions and frequencies of variations were compared for validation. The genomic positions of variations were determined by homology and frequencies were calculated by Jalview v2.10.5 consensus calculation algorithm (Waterhause et al., 2009).

### 2.2. Mutation analysis

Alignment results obtained using the software developed in this study were verified with the Nextstrain platform (Hadfield, 2018; Nextstrain, 2020^5^5NextStrain (2020). Nextstrain: analysis and visualization of pathogen sequence data [online] Website: https://nextstrain.org/ [accessed 17 May 2020].) that has been created to monitor virus evolution in real time. In this study, occupancy of sequences in alignment were considered in calculation of variation frequencies. Variations occurring over 10% frequency in clinical isolate genomes that have been downloaded into our database were evaluated and discussed specific to the genes. It must be noted that availability of these variations is also previously reported in other studies (Nextstrain, 2020; Pachetti et al., 2020; Wang et al., 2020; Phan et al., 2020) with different aspects. Despite the variations occurring over 10% frequency were discussed in the present study, it was noticed that SARS-CoV-2 isolate genomes were harbouring some of the variations over 20% frequency and causing missense mutations and so focused on these mutations in more detail and some charts were produced to show increasing EpiCoV entries, rate changes of the most frequent changes and rates of the most frequent mutations by continents.

The isolates with mistyped location and date annotations, nearly 2–3% of all downloaded entries, were ignored, while charts were created. Venn diagram presents simultaneously carried mutations and has been generated by Jvenn web tool (Bardou et al., 2014).

## 3. Results and discussion

### 3.1. Data optimization and development of Strategy Based Local Alignment tool: ODOTool

The alignments of randomly selected 200 SARS-CoV-2 isolate genome sequences were evaluated for validation according to genomic positions and frequencies of variations. It is well known that different datasets produce different results. As seen on Table 1, frequency calculation result by using both tools on the same dataset are consistent, showing validity of the results obtained by using the ODOTool. Further technical properties of ODOTool is excluded as these details are out of this Special Issue’s scopes. 

**Table 1:** Comparison of variation frequency calculations by ODOTool and MAFFT.

Reference genome position	Nucleotide exchange	Frequency (calculated by ODOTool)	Frequency (calculated by MAFFT)
241	C→T	77.0%	77.0%
1059	C→T	5.5%	5.5%
3037	C→T	82.5%	82.5%
11083	G→T	10.5%	10.5%
14408	C→T	82.5%	82.5%
14805	C→T	7.5%	7.5%
23403	A→G	82.0%	82.5%
25563	G→T	7.0%	7.0%
28881	G→A	58.0%	58.0%
28882	G→A	58.5%	58.5%
28883	G→C	58.5%	58.5%

In this study, 30366 SARS-CoV-2 isolate genome sequences were downloaded, standardised, aligned and in silico protein translation was performed. All processed data stored in a database for analysis. 

### 3.2. Mutation analysis

When the variations on SARS-CoV-2 genome is evaluated in general, several uneven used synonymous codons encode most of amino acids and this situation was defined as codon usage bias (CUB) that could have specific causes and consequences in different organisms (Belalov and Lukashev, 2013). CUB was explained by 2 primary circumstances; translational selection that means choosing the most suitable codon for translation and mutational pressure being gained by distinct probability of different substitution types like GC content, deoxycytidine methylation (C-phosphate-G), or subsequent deamination (C-T substitution) (Bulmer, 1987; Sharp et al., 1993; Belalov and Lukashev, 2013). 

Cytosine deamination has been identified as an important source of synonymous mutations (Duncan and Miller, 1980) managing the GC contents of RNA viruses (Pyrc et al., 2004). Because of cytosine deamination has been observed in all coronavirus genomes and proposed as a significant biochemical effect on coronavirus evolution (Woo et al., 2007), it is a predictable result that C-to T exchange has the most prominent numbers in all nucleotide change directions in SARS-CoV-2 genome.

In this study, 11 variations, with the incidence of over 10% have been detected in 30366 clinical SARS-CoV-2 isolate genomes (Table 2). It has been observed that 8 of these 11 variations; C1059T, G11083T, C14408T, A23403G, G25563T, G28881A, G28882A, and G28883C cause amino acid substitutions. Three variations that do not cause amino acid exchange were named as synonymous mutations in the literature (Kimura, 1977).

**Table 2:** Summary of variations evaluated in this article. Variations over 20% frequency are shown in bold * synonymous mutations.

Genome position	Nucleotide exchange	Frequency	Region	Amino acid exchange
241	C→T	70.99%	5’ UTR	-*
1059	C→T	18.10%	Nsp2/ORF1ab	T266I
3037	C→T	29.27%	Nsp3/ORF1ab	-*
11083	G→T	12.23%	Nsp6/ORF1ab	L36F
**14408**	**C→T**	**70.42%**	**Nsp12/ORF1ab**	**P323L**
14805	C→T	10.01%	Nsp12/ORF1ab	-*
**23403**	**A→G**	**70.47%**	**S Protein**	**D614G**
**25563**	**G→T**	**22.49%**	**ORF3a**	**Q57H**
**28881**	**G→A**	**26.30%**	**N Protein**	**R204K**
**28882**	**G→A**	**26.13%**	**N Protein**	
**28883**	**G→C**	**26.11%**	**N Protein**	**G205R**

When the 30366 SARS-CoV-2 genome sequences were aligned, the first encountered variation was observed to be C241T in the 5’ untranslated region (5’ UTR) being occurred nearly in 70% of SARS-CoV-2 isolates. Studies conducted on some viral genomes reported that the variations on UTRs may affect the activity, replication, and packaging of genomes, immune modulation and expression of genes (Silveria et al., 1995; De Lorenzo et al., 2016; Ng et al., 2017). This variation should be evaluated towards findings of these research studies with further in vitro studies as its frequency rate is 70.99% in whole genomes from all over the world. 

Rest of the 10 variations on nonstructural, structural, and accessory proteins are discussed below. 

#### 3.2.1. Variations on nonstructural proteins 

ORF1ab region of SARS-CoV-2 genome is an important polyprotein gene common in all CoVs and encodes 16 Nsps that include enzymes vital for the lifecycle of the virus, such as RNA depended polymerase, helicase and 3C-like proteinase (Brian and Baric, 2005; Gorblenya et al., 2006). Because of the critical role of these proteins on virulence and life cycle of the virus, some of these proteins have been proposed as potential target for antiviral therapy (Kwong et al., 2005; Briguglio et al., 2011; Zhou, 2020). Four variations over 10% were available on ORF1ab region. The first variation C3037T causes a synonymous mutation and seen in frequency of 29.3% in gene region encoding Nsp3 that is an important unit of the replication/transcription complex in CoVs (Lei et al., 2018). The other 3 variations, C1059T, G11083T, and C14408T causing amino acid substitutions are in gene regions of Nsp2, Nsp6, and Nsp12, respectively, in ORF1ab; with the incidence of over 10%. 

C1059T variation has been caused T266I amino acid exchange on Nsp2. But it is difficult to evaluate the effect of this exchange on the protein function, as the function of Nsp2 has not been resolved yet (Graham et al., 2005; Gadlage et al., 2008; Chen et al., 2020).

G11083T variation (reported previously by van Dorp et al., 2020) causing L36F exchange was present on Nsp6 gene region that is known with its role in inducing vesicles located around the microtubule regulation centre and ensuring membrane proliferation (Angelini et al., 2013). This variation was suggested as a homoplastic mutation (van Dorp et al., 2020) and suggested to be evaluated with these characteristics in further studies.

**Figure 2:**
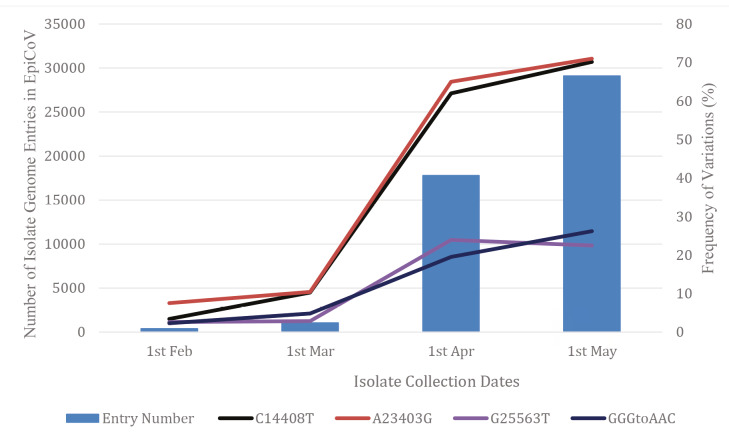
The increasing EpiCoV entries and rate changes of the most frequent variations by isolate collection dates.

C14408T (first reported by Pachetti et al., 2020) and C14805T found to be present on Nsp12 gene region with a frequency of 70.42% and 10.01% of isolates, respectively. Nsp12 is a vital protein for replication and pathogenesis and potential target for antiviral candidates in CoVs (Perlman and Netland, 2009; Wu et al., 2020; Yin and Wunderink, 2018). C14408T variation observed to be responsible from P323L exchange causing a missense mutation. The isolate genome sequence harbouring C14408T variation was first submitted to the GISAID from Lombardy, Italy, on February 20th, 2020 (EPI_ISL_412973), 20 days after the first COVID-19 case confirmed in Italy. Although C14805T (submitted on February 9th, 2020 England; EPI_ISL_412116) variation is rarer, it was emerged earlier than C14408T variation. Despite C14408T variation occurred later than C14805T, the incidence of this variation has increased sharply and reach over 70%, recently (Figure 2). This observation may be evaluated as a remarkable data about the effect of C14408T variation on the spread of the virus. 

#### 3.2.2 Variations on accessory and structural proteins

The increasing number of isolate genome entries in EpiCoV and rate changes of the most frequent variations by isolate collection dates are also given in Figure 2 for G25563T variation on accessory protein and A23403G variation on S protein and 3 consecutive variations (G28881A, G28882A, and G28883C) named as GGG to AAC on N protein. 

G25563T variation is on the gene region of Orf3a which is a unique membrane protein with its 3-membrane structure, the largest protein in the SARS related CoVs accessory protein family and is essential for the pathogenesis of the disease (Lu et al., 2010; Issa et al., 2020). G25560T variation also causes amino acid exchange of glutamine to histidine in residue 57 (Q57H).

There are also 4 structural proteins named as Spike (S), Envelope (E), Membrane (M), and Nucleocapsid (N) proteins and all has been encoded in all CoV genomes, including the SARS-CoV-2 (Brian and Baric, 2005; Gorblenya et al., 2020). In the current study, 4 variations were determined to cause amino acid substitutions in regions encoding S and N proteins with an incidence of over 10%.

A23403G variation is one of the most important variations that have been reported previously (Phan et al., 2020) caused D614G substitution on S protein. As in the C14408T of Nsp12 variation, A23403G variation of S protein is also available in 70.46% frequency in SARS-CoV-2 genomes isolated all over the world (Figure 2). S protein has important role in viral entry into the host cells (Gallagher and Buchmeiert, 2001). Viral entry and pathogenesis have been reported to be managed by a couple of mutations in its RBD of S protein in SARS-CoV (Wong et al., 2004; Li et al., 2005) aligned on residues between 331 and 524 for SARS-CoV-2 (Tai et al., 2020; Ou et al., 2020). RBD is located on the outer membrane of the virus and these properties of RBD make the S protein a suitable target for new treatment approaches (Tai et al., 2020) and the most of the ongoing protein subunit vaccine studies against SARS-CoV-2 (WHO, 2020b)^6^6WHO (2020b). Draft landscape of COVID-19 candidate vaccines [online]. Website: https://www.who.int/who-documents-detail/draft-landscape-of-covid-19-candidate-vaccines accessed 17 May 2020].. Therefore, any mutation on S protein of SARS-CoV-2 should be carefully tracked and evaluated, as this protein is the key target especially in the current vaccine development studies (Kiyotani et al., 2020).

Perhaps one of the most interesting SARS-CoV-2 variations occurred between genomic positions of 28881–28883. The GGG sequence of reference SARS-CoV-2 genome at these positions are converted to AAC in nearly 26% of rest of the isolate genomes. These 3 variations (G28881A, G28882A and G28883C) were seen simultaneously in about 99% of all isolates harbouring the variations. G28881A and G28882A exchanges cause R204K and G28883C exchange causes G205R amino acid substitutions on the N protein. N protein is an essential structural protein, playing very different roles in the regulation of infected cell metabolism and packaging of the viral genome important for both replication and transcription (Kang et al., 2020). 

**Figure 3:**
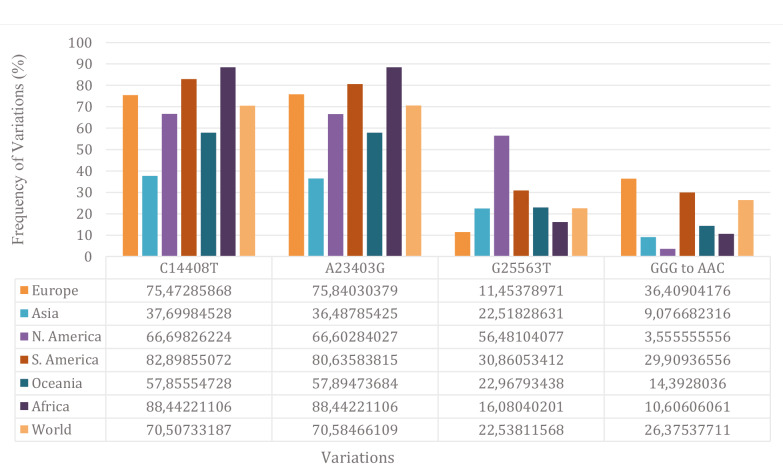
Rates of the most frequent variations by continents.

When the C14408T, G25563T, A23403G, and GGG to AAC variations with over 20% frequency were analysed, dramatic observations were seen in terms of appearance of these mutations in different continents (Figure 3). Figure 3 clearly shows that; G25563T mutation is developed by the isolates in N. America especially, GGG to AAC by the isolates in Europe, the same mutation is rare in N. America. C14408T and A23403G is common all over the world but especially in Africa, then S. America and this is followed by Europe (Figure 3). All these data could be used to trace diversity of virus in all over the world in combination with filiation studies. 

**Figure 4:**
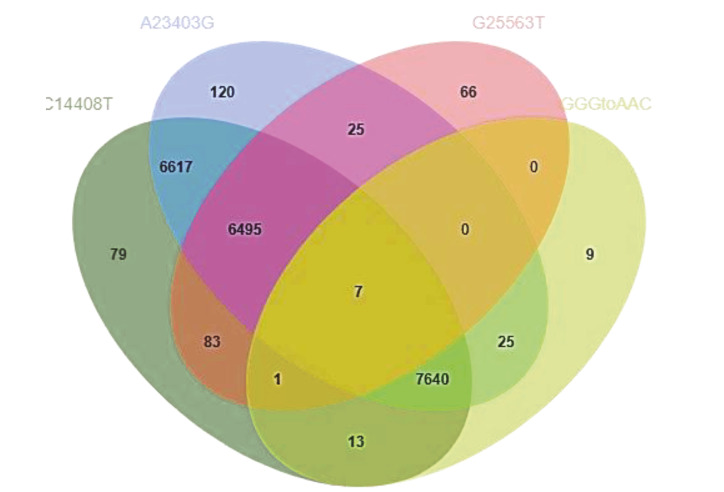
SARS-CoV-2 isolate numbers carrying the variations Green: Isolate numbers carrying C14408T, Blue: Isolate numbers carrying A23403G, Red: Isolate numbers carrying G25563T and Yellow: Isolate numbers carrying GGG to AAC variations between 28881–28883.

Our mutation analysis revealed that most of the isolates carry C14408T and A23403 variations simultaneously (Figure 4) in all continents (Figure 3). Nearly all isolates which carry G25563T, also carry C14408T and A23403G variations although their location distributions are not similar. Nearly no isolates carried GGG to AAC variations and G25563T variation simultaneously (Figure 4). The relation between these variations shown in Figure 4 may indicate that these mutations are coevoluating. Therefore, these results should be followed and evaluated by affiliation studies.

In conclusion; since the COVID-19 pandemic emerged globally, all the world has been searching ways to control the spread of the disease. As the virus’s adaptation strategies come from its nature, scientists put a massive effort on finding and development of suitable strategies for both effective prevention and treatment and on monitoring its virulence. Despite all these efforts, the virus continues to survive and spread, and this necessitates tracking any variations that occur on its genome to conduct further studies to find a way to control the disease. SARS-CoV-2 isolate genomes (30366) were aligned using the Strategy Based Local Alignment Tool (ODOTool) developed by our group and 11 variations in SARS-CoV-2 genome observed in over 10% of whole isolates from all over the world were discussed according to the date and location they occurred, the relationship with each other and their effect on the primary protein structure in the present study. Data were obtained as a result of evaluation of massive amount of genomic sequences in this study and expected to enlighten studies towards overcoming the SARS-CoV-2 infections.

## Acknowledgments 

This research received no specific grant from any funding agency in the public, commercial, or not for profit sectors. The authors declare that there is no conflict of interest. No ethics committee approval is necessary for this study. The genome sequences analysed in this study are available in the GISAID (https://www.gisaid.org/)^7^7GISAID (2020). Enabling rapid and open access to epidemic and pandemic virus data. [online] Website: https://www.gisaid.org/about-us/mission/ [ accessed 21 May 2020]. and GenBank (https://www.ncbi.nlm.nih.gov/nuccore/NC_045512)^8^8Genbank (2020). Accession No: NC_045512.2. Severe acute respiratory syndrome coronavirus 2 isolate Wuhan-Hu-1, complete genome [online]. Website: https://www.ncbi.nlm.nih.gov/nuccore/NC_045512 [accessed 29 May 2020]. repositories. Osman Mutluhan Uğurel and Oğuz Ata have made equal contribution in this study. The alignment of 30366 SARS-CoV-2 isolate genome sequences produced in this study will be publicly available for scientific use in http://www.dilekbalik.com/SARS-CoV-2_ODOTool/. This present paper should be referenced when the data in this alignment is used.
